# 伴JAK2 exon12突变与JAK2 V617F突变真性红细胞增多症患者的临床与实验室特征比较

**DOI:** 10.3760/cma.j.issn.0253-2727.2022.02.004

**Published:** 2022-02

**Authors:** 丹 刘, 培红 张, 泽锋 徐, 娇 马, 铁军 秦, 士强 曲, 秀娟 孙, 冰 李, 丽娟 潘, 玉娇 贾, 志坚 肖

**Affiliations:** 中国医学科学院血液病医院（中国医学科学院血液学研究所），实验血液学国家重点实验室，国家血液系统疾病临床医学研究中心，天津 300020 State Key Laboratory of Experimental Hematology, National Clinical Research Center for Blood Diseases, Institute of Hematology & Blood Diseases Hospital, Chinese Academy of Medical Science & Peking Union Medical College, Tianjin 300020, China

**Keywords:** 真性红细胞增多症, JAK2 exon12突变, 骨髓活组织病理形态, Polycythemia vera, JAK2 exon12 mutation, Bone marrow histology

## Abstract

**目的:**

比较伴JAK2 exon12与JAK2 V617F突变真性红细胞增多症（PV）患者的临床与实验室特征。

**方法:**

对2013年9月至2020年2月在中国医学科学院血液病医院就诊的570例符合WHO（2016）诊断分型标准且伴有JAK2基因突变的初诊PV患者进行回顾性分析，比较伴有JAK2 exon12与JAK2 V617F基因突变患者的临床与实验室特征。

**结果:**

①全部570例患者中，543例（95.3％）伴有单纯JAK2 V617F突变（JAK2 V617F组），24例（4.2％）伴有单纯JAK2 exon12突变（JAK2 exon12组），3例（0.5％）伴有JAK2 exon12与JAK2 V617F双突变。②27例伴有JAK2 exon12突变的患者中，JAK2 exon12突变类型包括缺失（10例，37.0％）、缺失伴插入（10例，37.0％）和错义突变（7例，25.9％）。③与JAK2 V617F组比较，JAK2 exon12组患者更年轻［中位年龄50（20～73）岁对59（25～91）岁，*P*＝0.040］，RBC［8.19（5.88～10.94）×10^12^/L对7.14（4.11～10.64）×10^12^/L，*P*<0.001］和红细胞比容［64.1％（53.7％～79.0％）对59.6％（47.2％～77.1％），*P*＝0.001］更高，而WBC［8.29（3.20～18.99）×10^9^/L对12.91（3.24～38.30）×10^9^/L，*P*<0.001］、PLT［313（83～1433）×10^9^/L对470（61～2169）×10^9^/L，*P*<0.001］和红细胞生成素［0.70（0.06～3.27）U/L对1.14（0.01～10.16）U/L，*P*＝0.002］更低。④选取性别、年龄匹配的JAK2 exon12组与JAK2 V617F组各20例患者的骨髓活组织病理标本，JAK2 exon12组巨核细胞疏松成簇的患者比例显著低于JAK2 V617F组（40.0％对80.0％，*P*＝0.022），其他骨髓病理形态特性相似。⑤JAK2 exon12组、JAK2 V617F突变组的中位随访时间分别为30（4～83）个月、37（1～84）个月，两组总生存（*P*＝0.422）和无血栓生存（*P*＝0.900）差异无统计学意义。

**结论:**

JAK2 exon12突变患者较JAK2 V617F突变患者更为年轻，红系增生更显著，骨髓巨核细胞疏松成簇更少见。

## Abstract (en)

**Objective:**

To compare clinical and laboratory features between JAK2 exon12 and JAK2 V617F mutated polycythemia vera (PV).

**Methods:**

We collected data from 570 consecutive newly-diagnosed subjects with PV and JAK2 mutation, and compared clinical and laboratory features between patients with JAK2 exon12 and JAK2 V617F mutation.

**Results:**

543（95.3％）subjects harboured JAK2 V617F mutation (JAK2 V617F cohort), 24（4.2％）harboured JAK2 exon12 mutations (JAK2 exon12 cohort), and 3（0.5％）harboured JAK2 exon12 and JAK2 V617F mutations. The mutations in JAK2 exon12 including deletion (*n*＝10, 37.0％), deletion accompanied insertion (*n*＝10, 37.0％), and missense mutations (*n*＝7, 25.9％). Comparing with JAK2 V617F cohort, subjects in JAK2 exon12 cohort were younger［median age 50（20–73）years versus 59（25–91）years, *P*＝0.040］, had higher RBC counts［8.19（5.88–10.94）×10^12^/L versus 7.14（4.11–10.64）×10^12^/L, *P*<0.001］and hematocrit［64.1％（53.7–79.0％）versus 59.6％（47.2％–77.1％）, *P*＝0.001］, but lower WBC counts［8.29（3.2–18.99）×10^9^/L versus 12.91（3.24–38.3）×10^9^/L, *P*<0.001］, platelet counts［313（83–1433）×10^9^/L versus 470（61–2169）×10^9^/L, *P*<0.001] and epoetin［0.70（0.06–3.27）versus 1.14（0.01–10.16）IU/L, *P*＝0.002］levels. We reviewed bone marrow histology at diagnosis in 20 subjects with each type of mutation matched for age and sex. Subjects with JAK2 exon12 mutations had fewer loose megakaryocyte cluster (40％ versus 80％, *P*＝0.022) compared with subjects with JAK2 V617F. The median follow-ups were 30 months (range 4–83) and 37 months (range 1–84) for cohorts with JAK2 V617F and JAK2 exon12, respectively. There was no difference in overall survival (*P*＝0.422) and thrombosis-free survival (*P*＝0.900).

**Conclusion:**

Compared with patients with JAK2 V617F mutation, patients with JAK2 exon12 mutation were younger, and had more obvious erythrocytosis and less loose cluster of megakaryocytes.

真性红细胞增多症（PV）是一种费城染色体阴性的骨髓增殖性肿瘤（MPN），95％以上的PV患者伴有JAK2突变（JAK2 exon12和JAK2 V617F）[1]–[2]，约3％的PV患者伴有JAK2 exon12突变[3]–[4]。既往研究发现伴有JAK2 exon12突变与伴有JAK2 V617F突变的PV患者临床特征存在差异，表现为前者更为年轻、红细胞或血红蛋白水平更高[3],[5]–[9]，但两组患者骨髓活组织检查病理形态特征是否有显著差异目前尚不明确。本研究回顾性分析了新诊断伴有JAK2基因突变的570例PV患者，比较了JAK2 exon12与JAK2 V617F突变患者的临床和实验室特征（包括骨髓活组织检查病理形态特征）。

## 病例与方法

1. 病例：纳入本研究的病例为2013年9月至2020年2月在中国医学科学院血液病医院新诊断的PV患者。纳入标准：①符合世界卫生组织（WHO）2016版诊断分型标准[10]；②有确诊时完整的临床及实验室检查数据；③伴有JAK2 exon12和（或）JAK2 V617F突变；④年龄≥18岁。共570例患者纳入本研究，其中543例（95.3％）患者伴有单纯JAK2 V617F突变（JAK2 V617F组），24例（4.2％）伴有单纯JAK2 exon12突变（JAK2 exon12组），3例（0.5％）伴有JAK2 exon12与JAK2 V617F双突变。

2. JAK2基因突变及二代测序检测：JAK2 V617F突变负荷（VAF）采用实时荧光定量PCR[11]或二代测序（NGS）进行定量检测，或采用琼脂糖凝胶电泳进行定性检测。JAK2 exon12突变采用sanger测序进行定性检测或NGS进行定量检测[3]。NGS测序方法见参考本研究组此前报道[12]，检测基因组套涵盖112个血液肿瘤相关基因，用PCR引物扩增目的基因组，富集后采用Ion Torrent半导体测序平台进行测序[12]。平均基因覆盖率98.1％，平均测序深度1310×。

3. 骨髓活组织病理切片再分析：我们重新分析了性别、年龄匹配的伴有单纯JAK2 exon12或JAK2 V617F突变各20例患者，以及3例JAK2 exon12与JAK2 V617F双突变患者骨髓活组织病理切片。主要分析参数详见本研究组此前报道[13]。骨髓纤维化分级的评估参照欧洲MF分级共识标准[14]。

4. 随访：随访截止日期为2020年10月30日，随访资料来源于住院、门诊病历及电话随访记录。

5. 治疗方案及疗效评估：年轻患者（<60岁）首选重组干扰素（IFN）治疗，IFN起始剂量为300万单位每周3次；年龄≥60岁或不能耐受IFN的患者应用羟基脲（HU）治疗，HU起始剂量为20 mg·kg^−1^·d^−1^。除外失访患者，根据患者初始治疗方案分组，JAK2 exon12突变组42.9％（9/21）的患者接受HU治疗，42.9％（9/21）接受IFN治疗，9.5％（2/21）接受IFN+HU治疗，4.8％（1/21）接受芦可替尼治疗；JAK2 V617F组53.6％（253/472）的患者接受HU治疗，17.2％（81/472）接受IFN治疗，24.4％（115/472）接受HU+IFN治疗，0.2％（1/472）接受芦可替尼治疗，4.6％（22/470）未接受降细胞治疗。血液学疗效判断标准见本研究组此前报道[15]。

6. 统计学处理：偏态分布计量资料以中位数表示，组间比较采用非参数Mann-Whitney *U*检验。率的比较采用卡方检验或Fisher确切概率法。相关性分析采用Spearman检验。总生存期（OS）定义为确诊日期至死亡日期或确诊日期至最后随访日期。无血栓生存期定义为确诊至确诊后首次发生血栓、或确诊日期至死亡、或确诊至最后随访日期。生存分析采用Kaplan-Meier方法，生存曲线比较采用Log-rank检验，多因素分析采用Cox回归模型。*P*<0.05被认为差异具有统计学意义。所有数据采用SPSS 26.0软件进行数据处理。

## 结果

1. 基因突变特征：27例伴有JAK2 exon12突变的患者中，其中3例伴有JAK2 exon12与JAK2 V617F双突变。JAK2 exon12突变类型包括缺失（10例，37.0％），缺失伴插入（10例，37.0％），错义突变（7例，25.9％）。图1展示了27例JAK2 exon12突变阳性患者的突变位点，最常见的突变位点为p.N542-E543del（6例，22.2％），其次为p.K539L（5例，18.5％）。7例JAK2 exon12突变患者进行NGS检测，其中3例患者伴有JAK2外其他基因突变（1例伴有KMT2D和DNMT3A突变，1例伴有KMT2D和NOTCH4突变，1例伴有SETD2突变）。该7例患者JAK2 exon12 VAF中位数为27％（11％～31％）。

**图1:**
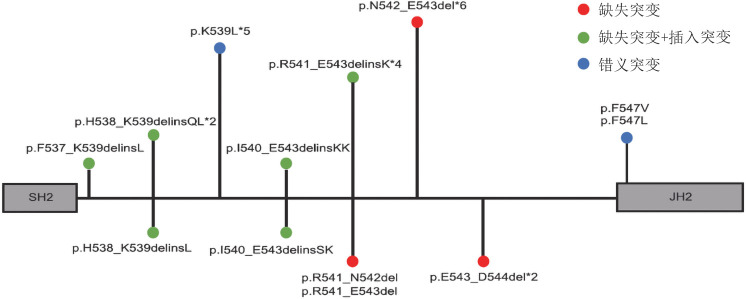
27例伴JAK2 exon12突变真性红细胞增多症患者基因突变图谱 SH2：Src同源结构域；JH2：酪氨酸激酶的假激酶结构域

JAK2 V617F组患者确诊时JAK2 V617F VAF中位数为47.6％（1.1％～96.7％）。116例JAK2 V617F组患者进行NGS检测，54例（46.6％）伴有JAK2外其他基因突变，其中最常见的突变基因为TET（24例，20.7％），其次为DNMT3A（7例，6.0％）。

2. 临床及实验室特征：与JAK2 V617F组比较，JAK2 exon12组年龄较低［中位年龄50（20～73）岁对59（25～91）岁，*P*＝0.026］，RBC［8.19（5.88～10.94）×10^12^/L对7.14（4.11～10.64）×10^12^/L，*P*<0.001］和红细胞比容（HCT）［64.1％（53.7％～79.0％）对59.6％（47.2％～77.1％），*P*＝0.001］较高，而WBC［8.29（3.20～18.99）×10^9^/L对12.91（3.24～38.30）×10^9^/L，*P*<0.001］、PLT［313（83～1433）×10^9^/L对470（61～2169）×10^9^/L，*P*<0.001］、红细胞生成素（EPO）［0.70（0.06～3.27）U/L对1.14（0.01～10.16）U/L，*P*＝0.002］较低，JAK2 exon12组WBC>10×10^9^/L、PLT>300×10^9^/L的患者比例均低于JAK2 V617F组［29.2％（7/24）对76.2％（413/542），*P*<0.001；50.0％（12/24）对83.6％（453/542），*P*<0.001］（表1）。

JAK2 exon12组与JAK2 V617F组比较，女性［45.8％（11/24）对48.1％（261/543），*P*＝0.839］、左肋缘下可触及脾脏［30.0％（6/20）对25.2％（130/515），*P*＝0.607］、确诊前发生血栓［18.2％（4/22）对30.4％（162/533），*P*＝0.247］、存在体质性症状［9.1％（2/22）对3.1％（16/522），*P*＝0.162］的患者占比差异没有统计学意义（表1）。

**表1:** 单纯JAK2 exon12与JAK2 V617F突变真性红细胞增多症（PV）患者确诊时临床特征比较

临床特征	JAK2 exon12突变（24例）	JAK2 V617F突变（543例）	*P*值
年龄［岁，*M（*范围）］	50（20～73）	59（25～91）	0.026
女性［例（％）］	11（45.8）	261（48.1）	0.839
脾脏左缘肋缘下可触及［％（阳性例数/检查例数）］	30.0（6/20）	25.2（130/515）	0.607
确诊前血栓史［％（阳性例数/检查例数）］	18.2（4/22）	30.4（162/533）	0.247
动脉血栓	18.2（4/22）	28.9（154/533）	0.342
脑梗死	18.2（4/22）	23.6（126/533）	0.622
心肌梗死	0（0/22）	5.8（31/533）	0.393
其他	0（0/22）	1（6/533）	1.000
静脉血栓	4.5（1/22）	2.8（15/533）	1.000
体质性症状［％（阳性例数/检查例数）］	9.1（2/22）	3.1（16/522）	0.162
盗汗	0（0/22）	1.3（7/522）	1.000
发热	0（0/22）	0（0/522）	1.000
体重减轻	9.1（2/22）	2.1（11/522）	0.093
皮肤瘙痒［％（阳性例数/检查例数）］	0（0/22）	3.4（18/522）	0.070
HGB［g/L，*M*（范围）］	194（162～241）	195（150～254）	0.664
RBC［×10^12^/L，*M*（范围）］	8.19（5.88～10.94）	7.14（4.11～10.64）	<0.001
HCT［％，*M*（范围）］	64.1（53.7～79.0）	59.6（47.2～77.1）	0.001
WBC［×10^9^/L，*M*（范围）］	8.29（3.20～18.99）	12.91（3.24～38.3）	<0.001
WBC>10×10^9^/L［％（阳性例数/检测例数）］	29.2（7/24）	76.2（413/542）	<0.001
PLT［×10^9^/L，*M*（范围）］	313（83～1433）	470（61～2169）	<0.001
PLT>300×10^9^/L［％（阳性例数/检测例数）］	50.0（12/24）	83.6（453/542）	<0.001
EPO［U/L，*M（*范围）］	0.70（0.06～3.27）	1.14（0.01～10.16）	0.002
染色体核型异常［％（阳性例数/检测例数）］	0（0/20）	4.6（20/432）	0.616

注：HCT：红细胞比容；EPO：红细胞生成素

452例患者确诊时染色体核型可供分析，JAK2 exon12组与JAK2 V617F组确诊时伴有染色体核型异常的患者比例分别为0（0/20）、4.6％（20/432）（*P*＝0.616）。

JAK2 exon12组伴缺失、缺失伴插入或错义突变患者的年龄、性别、脾脏左肋缘下可触及的患者比例、外周血细胞计数、EPO水平差异均没有统计学意义（表2）。

**表2:** 不同JAK2 exon12突变类型患者的临床及骨髓活组织检查病理特征比较（除外3例JAK2 exon12与JAK2 V617F双突变患者）

指标	错义突变（6例）	缺失突变（9例）	缺失突变+插入突变（9例）	*P*值
年龄［岁，*M*（范围）］	38（24～60）	49（34～73）	62（20～72）	0.170
女性［％（阳性例数/检测例数）］	66.7（4/6）	22.2（2/9）	55.6（5/9）	0.262
脾脏左肋缘下可触及［％（阳性例数/检测例数）］	60.0（3/5）	25.0（2/8）	14.3（1/7）	0.283
确诊前血栓史［％（阳性例数/检测例数）］	0（0/5）	33.3（3/9）	12.5（1/8）	0.395
存在体质性症状［％（阳性例数/检测例数）］	0（0/5）	11.1（1/9）	12.5（1/8）	1.000
HGB［g/L，*M*（范围）］	210（167～241）	194（174～222）	193（162～230）	0.605
RBC［×10^12^/L，*M*（范围）］	7.76（6.91～10.94）	8.74（6.92～9.09）	8.10（5.88～10.31）	0.781
HCT［％，*M*（范围）］	62.7（53.7～79.0）	63.4（57.2～73.4）	65.3（56.7～72.2）	0.989
WBC［×10^9^/L，*M*（范围）］	9.41（3.24～18.99）	6.27（5.32～9.29）	8.73（3.42～16.37）	0.407
PLT［×10^9^/L，*M*（范围）］	285（131～1433）	367（153～636）	250（80～471）	0.685
EPO［U/L，*M*（范围）］	0.99（0.08～2.80）	0.61（0.06～0.79）	0.99（0.47～3.27）	0.176
粒系增生程度增高［％（阳性例数/检测例数）］	80.0（4/5）	57.1（4/7）	50.0（4/8）	0.728
粒系核左移［％（阳性例数/检测例数）］	60.0（3/5）	14.3（1/7）	37.5（3/8）	0.298
红系增生程度增高［％（阳性例数/检测例数）］	80.0（4/5）	85.7（6/7）	100.0（8/8）	0.495
红系核左移［％（阳性例数/检测例数）］	0（0/5）	14.3（1/7）	37.5（3/8）	0.387
巨核细胞增生程度增高［％（阳性例数/检测例数）］	80.0（4/5）	71.4（5/7）	75.0（6/8）	1.000
巨核细胞疏松成簇［％（阳性例数/检测例数）］	80.0（4/5）	42.9（3/7）	12.5（1/8）	0.540
巨核细胞密集成簇［％（阳性例数/检测例数）］	20.0（1/5）	14.3（1/7）	0（0/8）	0.495

注：HCT：红细胞压积；EPO：红细胞生成素

JAK2 V617F组患者JAK2 V617F VAF与患者年龄（*r*＝0.193）、WBC（*r*＝0.459）、RBC（*r*＝0.282）、HGB（*r*＝0.218）、HCT（*r*＝0.303）、乳酸脱氢酶（*r*＝0.492）呈正相关（*P*值均<0.001），而与PLT呈负相关（*r*＝−0.173，*P*<0.001）。

比较JAK2 exon12组不同突变类型患者与JAK2 V617F组的临床及实验室特征，发现伴有JAK2 exon12错义突变组中位年龄显著低于JAK2 V617F组［38（24～60）岁对59（25～91）岁，*P*＝0.007］，其他临床、实验室特征与JAK2 V617F组比较差异无统计学意义（*P*>0.05），这可能与错义突变组患者例数较少有关。伴有JAK2 exon12缺失或缺失伴插入突变组中位年龄与JAK2 V617F组差异无统计学意义［49（34～73）岁对59（25～91）岁，*P*＝0.174；62（20～72）岁对59（25～91）岁，*P*＝0.915］，但WBC显著低于JAK2 V617F组［6.27（5.32～9.29）×10^9^/L对12.91（3.24～38.30）×10^9^/L，*P*<0.001；8.73（3.42～16.37）×10^9^/L对12.91（3.24～38.30）×10^9^/L，*P*＝0.012］、PLT显著低于JAK2 V617F组［367（153～636）×10^9^/L对470（61～2169）×10^9^/L，*P*＝0.017；250（80～471）×10^9^/L对470（61～2169）×10^9^/L，*P*＝0.003］，HCT显著高于JAK2 V617F组［63.4％（57.2％～73.4％）对59.6％（47.2％～77.1％），*P*＝0.029；65.3％（56.7％～72.2％）对59.6％（47.2％～77.1％），*P*＝0.045］。JAK2 exon12缺失突变组RBC显著高于JAK2 V617F组［8.74（6.92～9.09）×10^12^/L对7.14（4.11～10.64）×10^12^/L，*P*＝0.001］。

3. 骨髓病理形态特征：在JAK2 exon12组和JAK2 V617F组中分别选取20例性别、年龄匹配的患者，比较两组骨髓活组织病理特征，结果见表2。JAK2 exon12组与JAK2 V617F组患者骨髓增生程度没有显著差异［中位数75％（50％～90％）对75％（60％～90％），*P*＝0.968］，红系增多（90％对100％，*P*＝0.487）、巨核细胞增多（75％对95％，*P*＝0.182）的患者比例差异无统计学意义，JAK2 exon12组粒系增生程度增高的患者更少（60％对90％，*P*＝0.065）。两组患者均可见胞体大、分叶多的异常巨核细胞，但JAK2 exon12组存在巨核细胞疏松成簇的患者比例显著低于JAK2 V617F组（40％对80％，*P*＝0.022），而两组存在巨核细胞密集成簇的患者比例差异无统计学意义（10％对20％，*P*＝0.661，表3）。

JAK2 exon12组与JAK2 V617F组患者骨髓纤维化分级（*P*＝0.278）、血窦增宽（100％对80％，*P*＝0.106）、窦内造血细胞增多（100％对85％，*P*＝0.231）、嗜酸细胞增多（10％对30％，*P*＝0.235）、淋巴细胞灶（10％对10％，*P*＝1.000）的患者比较差异均无统计学意义（表3）。

**表3:** 年龄、性别匹配的JAK2 exon12与JAK2 V617F突变真性红细胞增多症患者确诊时骨髓病理特征比较

病理特征	JAK2 exon12突变（20例）	JAK2 V617F突变（20例）	*P*值
骨髓增生程度［％，*M*（范围）］	75（50～90）	75（60～90）	0.968
粒系增生［％（例）］			0.065
增高	60.0（12）	90.0（18）	
正常	40.0（8）	10.0（2）	
粒系核左移［％（例）］	35.0（7）	40.0（8）	1.000
红系增生［％（例）］			0.487
增高	90.0（18）	100.0（20）	
正常	10.0（2）	0.0（0）	
红系核左移［％（例）］	20.0（4）	25.0（5）	1.000
粒红比例［％（例）］			0.189
增高	5.0（1）	5.0（1）	
正常	15.0（3）	30.0（6）	
减低	80.0（16）	65.0（13）	
巨核细胞增生［％（例）］			0.182
增高	75.0（15）	95.0（19）	
正常	25.0（5）	5.0（1）	
胞体大分叶多巨核［％（例）］	70.0（14）	90.0（18）	0.235
巨核细胞疏松成簇	40.0（8）	80.0（16）	0.022
巨核细胞密集成簇	10.0（2）	20.0（4）	0.661
骨髓纤维化分级［％（例）］			0.278
MF-0级	25.0（5）	50.0（10）	
MF-1级	60.0（12）	35.0（7）	
MF-2级	15.0（3）	15.0（3）	
血窦增宽［％（例）］	100.0（20）	80.0（16）	0.106
窦内造血细胞增多［％（例）］	100.0（20）	85.0（17）	0.231
嗜酸细胞增多［％（例）］	10.0（2）	30.0（6）	0.235
淋巴细胞灶［％（例）］	10.0（2）	10.0（2）	1.000

JAK2 exon12缺失、缺失伴插入、错义突变患者的骨髓病理形态特征差异无统计学意义（表2）。伴JAK2 exon12缺失或缺失伴插入突变的患者存在疏松巨核细胞的患者比例显著低于JAK2 V617F组［42.9％（3/7）对80.0％（16/20），*P*＝0.043；12.5％（1/8）对80.0％（16/20），*P*＝0.001］，而伴JAK2 exon12错义突变的患者骨髓病理形态特征与JAK2 V617F组差异无统计学意义（*P*值均>0.05，表2）。

4. JAK2 V617F与exon12双突变患者临床及实验室特征：3例JAK2 exon12与JAK2 V617F突变双阳性患者，女性2例，男性1例。JAK2 exon12突变位点分别为p.H538_K539delinsQL、p.K539L、p.E543_D544del。患者中位确诊年龄为69（55～70）岁，JAK2 V617F VAF均较低（2.5％～15.7％），中位RBC为10.37（7.62～10.71）×10^12^/L，中位HGB为200（183～223）g/L，中位WBC为10.74（2.67～11.18）×10^9^/L，中位PLT为489（101～687）×10^9^/L。3例患者染色体核型均正常，2例患者骨髓活组织检查网状纤维染色为MF-2级，1例患者为MF-1级。1例患者确诊15个月后转为急性髓系白血病（AML）。

伴有单纯JAK2 exon12、单纯JAK2 V617F及JAK2 exon12和JAK2 V617F双突变患者的骨髓活组织检查病理形态见图2。

**图2:**
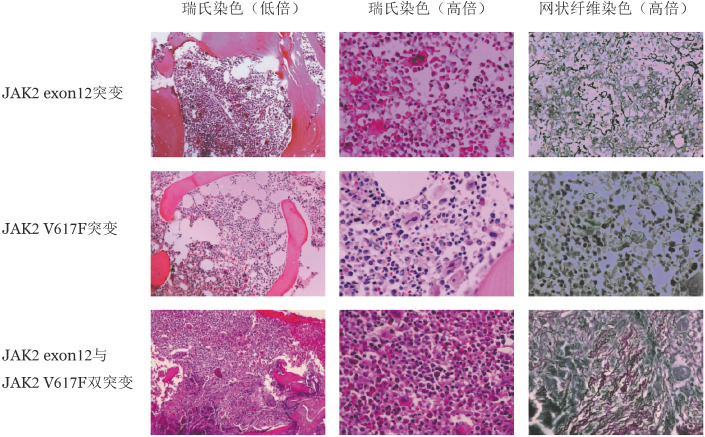
不同类型JAK2基因突变的真性红细胞增多症患者骨髓活组织病理形态特征

5. 生存比较：JAK2 exon12组3例（12.5％）患者失访，JAK2 V617F组60例（11.0％）患者失访。除外失访患者，JAK2 exon12组中位随访时间30（4～83）个月，JAK2 V617F突变组中位随访时间37（1～84）个月。随访期间JAK2 exon12组没有患者死亡，3年累积死亡率为0；JAK2 V617F组15例患者死亡，3年累积死亡率为3.1％（95％*CI* 2.2％～4.0％），两组OS差异没有统计学意义（*P*＝0.422，图3A）。经年龄、治疗方案调整后，两组患者OS差异也没有统计学意义（*P*＝0.989）。

JAK2 exon12组1例患者确诊后发生血栓，3年累积血栓发生率为5.9％（95％*CI* 0.2％～11.2％）；JAK2 V617F组25例患者确诊后发生血栓，3年累积血栓发生率为4.3％（95％*CI* 3.2％～4.4％），两组患者无血栓生存差异没有统计学意义（*P*＝0.900，图3B），经年龄、治疗方案调整后，两组患者无血栓生存差异也无统计学意义（*P*＝0.983）。

**图3:**
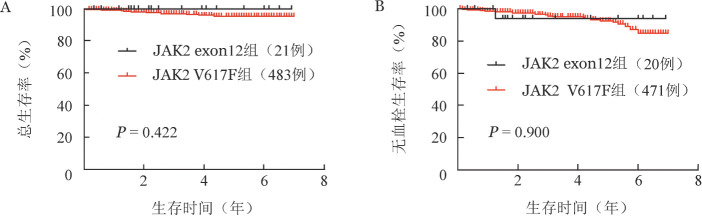
JAK2 exon12突变与JAK2 V617F突变真性红细胞增多症患者生存曲线（A：总生存；B：无血栓生存）

评估随访期间最佳血液学疗效，33.5％（190/333）例患者获得完全血液学反应（CHR）。获得CHR患者5年累积死亡率为1.3％（95％*CI* 0～1.3％），未获得CHR患者5年累积死亡率为3.0％（95％*CI* 0.8％～5.2％），两组患者OS差异无统计学意义（*P*＝0.528）。

## 讨论

既往关于伴有JAK2 exon12突变PV患者的研究报道主要是西方国家患者数据[3]–[9],[16]–[18]，PV患者JAK2 exon12检出率为3％～5％。亚洲地区患者仅有2篇文献报道：中国台湾一项研究报道22例PV患者，17例（77％）伴有JAK2 V617F突变，5例（23％）伴有JAK2 exon12突变，研究者认为亚洲PV患者JAK2 exon12突变检出率更高[19]；韩国一项研究报道42例PV患者，其中37例（88％）伴有JAK2 V617F突变，12例（12％）伴有JAK2 exon12突变，研究者也得出相似结论：韩国的PV患者中JAK2 exon12突变者比例更高[20]。但上述两项研究纳入患者例数均较少，本研究纳入570例PV患者，伴JAK2 exon12突变的患者占4.7％，与既往西方国家患者数据一致[3]–[9],[16]–[18]，提示亚洲PV患者伴JAK2 exon12突变的患者比例并不高于西方国家的PV患者。

本研究结果提示与JAK2 V617F突变患者比较，JAK2 exon12突变患者确诊时更年轻，外周血以红细胞增高为主，而WBC、PLT及EPO水平低于JAK2 V617F突变患者，两组脾脏肿大的患者比例没有显著差异，与既往研究结果一致[3],[8]–[9]。JAK2 exon12突变的小鼠也表现为单纯红细胞增高，RBC、HGB显著高于野生型或JAK2 V617F突变小鼠，而PLT、WBC水平与野生型小鼠无显著差异，均显著低于JAK2 V617F突变小鼠[21]。

既往仅有小样本研究报道JAK2 exon12突变的PV患者骨髓活组织检查病理形态特征，本研究比较JAK2 exon12与JAK2 V617F突变患者骨髓病理特征，结果提示JAK2 exon12突变患者骨髓多为单纯红系增生，部分患者骨髓红系、粒系、巨核系三系增生程度均增高，可见胞体大、分叶多的异常巨核细胞，与既往研究结果一致[3],[5],[8],[19],[22]。本研究发现JAK2 exon12突变患者存在巨核细胞疏松成簇的患者比例显著低于JAK2 V617F突变患者，这可能与JAK2 exon12突变患者巨核系增生程度低于JAK2 V617F突变患者相关，但目前并不清楚巨核细胞疏松成簇是否与两种不变类型的发病机制存在差异相关。存在巨核细胞疏松成簇对患者OS、无血栓生存、无骨髓纤维化生存并无显著影响，但这需要延长随访时间、扩大样本量来验证。

本研究报道了3例同时伴有JAK2 exon12与JAK2 V617F突变的PV患者，其中2例患者确诊时骨髓MF-2级，1例患者确诊后不久转为AML，提示双突变的患者可能疾病进展更快，这也提示确诊疾病时同时进行包含JAK2 V617F与JAK2 exon12突变的检测可能是必要的，能发现更高危的患者。但本研究仅3例JAK2 exon12与JAK2 V617F双突变患者，双突变的临床意义需要更大样本研究来验证。

Tefferi等[9]研究提示JAK2 exon12突变的PV患者OS优于JAK2 V617F突变患者，但校正年龄和（或）WBC后，两组患者OS差异没有统计学意义。本研究结果提示JAK2 exon12与JAK2 V617F突变患者OS没有统计学差异，可能与本研究随访时间较短相关。因随访时间较短，单纯JAK2 exon12突变与JAK2 V617F突变两组患者均未出现疾病进展为PV后骨髓纤维化或AML患者，两组患者疾病进展的风险是否存在差异还需要延迟随访时间、扩大样本量来验证。本研究中患者确诊前血栓总发生率为30.1％（168/558），稍高于既往研究报道（约25％）[23]–[24]，这可能与中国健康体检普及率低于西方国家，患者就诊晚，部分患者出现血栓症状才就诊相关。

生理状态下，EPO结合EPO受体（EpoR），使JAK2形成同源二聚体及自体磷酸化，激活下游的信号通路，包括STAT5、PI3K及MAPK信号通路，从而促进骨髓细胞增殖、分化，抑制凋亡[25]。而JAK2突变的PV患者可不依赖EPO，持续使JAK2形成同源二聚体及自体磷酸化，从而激活下游信号通路，导致细胞过度增殖、分化[25]，引起骨髓增生程度增高，外周血细胞增多。既往研究提示，JAK2 exon12突变的小鼠骨髓中多能祖细胞（MPP）和巨核-红系祖细胞（MEP）的比例显著高于野生型或JAK2 V617F突变小鼠；骨髓细胞体外培养，红系集落形成能力也显著高于野生型或JAK2 V617F突变小鼠[21]。此外，JAK2 exon12突变的小鼠骨髓细胞肝铁调素（hepcidin）表达低于野生型或JAK2 V617F突变小鼠，而红富铁素（ERFE）表达更高，提示JAK2 exon12突变可能通过调节铁代谢而更利于红细胞增殖[21]。本研究组发现JAK2 exon12突变的PV患者确诊时缺铁较JAK2 V617F突变患者更显著[26]，提示铁代谢的改变可能也是JAK2 exon12突变PV患者发病机制之一。但JAK2 exon12突变与JAK2 V617F突变的PV患者发病机制还需要更多的机制研究。

本研究具有以下局限性：单中心回顾性研究，随访时间较短，进行NGS测序的患者例数较少，JAK2 exon12与JAK2 V617F突变患者临床、实验室及预后特征比较没有排除其他基因突变的影响。
